# Evaluation of the Performance of New Fluorescence Immunoassay POCTs for Determining the Value of Vitamin D in Whole Blood

**DOI:** 10.3390/molecules31010130

**Published:** 2025-12-30

**Authors:** Alice Palermiti, Alessandra Manca, Fabrizio Mastrantonio, Domenico Maiese, Elena Cat Genova, Giorgia Menegatti, Marco Simiele, Camilla Martino, Amedeo De Nicolò, Antonio D’Avolio

**Affiliations:** 1 Laboratory of Clinical Pharmacology and Pharmacogenetics, Department of Medical Sciences, University of Turin, 10149 Turin, Italy; 2 A. Menarini Diagnostics, 50131 Florence, Italy; 3 CoQua Lab s.r.l., 10149 Turin, Italy

**Keywords:** 25-OH-VD, quantitation technique, point of care test

## Abstract

VD (VD), a hormone-like, fat-soluble molecule, is essential for several biological processes, such as gene regulation, calcium balance, bone health, immune function, antiviral defense, and neuromuscular activity. Its deficiency is associated with various disorders, including chronic hypocalcemia and increased risk of severe diseases, such as COVID-19. Monitoring 25-hydroxyVD (25-OH-D) levels is vital, with serum 25-OH-VD being the standard marker. While chromatography and immunometric assays are well-established, innovative point-of-care (POC) platforms like AFIAS-1^®^ (Boditech & Menarini, Gangwon, Republic of Korea) are emerging as rapid and automated alternatives, particularly advantageous for decentralized settings such as pharmacies, general practitioners’ offices, and specialized hospital centers like intensive care units. This study compared AFIAS-1^®^ using whole blood with the gold standard UHPLC-MS/MS using plasma in 50 samples, showing a strong correlation and confirming AFIAS-1^®^ as a reliable method for measuring 25-OH-D levels.

## 1. Introduction

From a biochemical perspective, Vitamin D (VD) is a group of closely related compounds that share a common precursor, 7-dehydrocholesterol, also known as pre-VD. In humans, the most important compounds in this category are vitamins D2 (ergocalciferol) and D3 (cholecalciferol) [1]: while VD2 is acquired from diet and vitamin supplements, VD3 is only partially acquired from diet/supplements, being mostly generated by the skin after UVB light exposure [2,3,4]. VD2 and D3 are hydroxylated in the liver to produce 25-OH-VD, which is subsequently further hydroxylated in the kidneys to produce 1,25-(OH)_2_-VD. Although 1,25-(OH)_2_-VD2 is the biologically active form of VD, a patient’s overall VD status is determined by their total 25-OH-VD levels (sum of 25-OH-VD2 and 25-OH-VD3), [5,6,7,8,9,10], which represent their circulating VD reservoir.

In the general population, individuals’ 25-OH-VD concentrations should fall within the range of 30 to 50 ng/mL (75 to 125 nmol/L) [11].

Furthermore, testing VD is essential, since many patients have severe VD insufficiency [25-OH-VD < 10 ng/mL] [12,13].

The number of studies on VD has grown significantly during the last few decades. Many acute and chronic conditions, including bone metabolism disorders (problems in calcium/phosphate homeostasis), kidney and cardiovascular diseases, obesity, cancer, autoimmune diseases, diabetes, infectious diseases, and aging, are now directly linked to VD deficiency, as reported in the scientific literature [14,15,16].

In this context, the need for precise VD testing in clinical settings and personalized VD supplementation for all medical diseases has more than doubled in the last ten years, especially for critically sick patients who are admitted to the intensive care unit [17,18].

VD deficiency is very common in the intensive care unit, and several epidemiological studies have linked VD deficiency to a variety of diseases in various organ systems [14]. In particular, a UK Biobank study examined associations between VD deficiency, biomarkers of systemic inflammation, and specific mortality. The findings suggest that low VD levels are associated with increased risk of mortality, indicating a potential link between VD deficiency and disease in multiple organ systems.

A retrospective study by Venkatram et al. [16] examined 437 patients admitted to medical intensive care. The results showed that 77.8% of patients had VD deficiency, and this condition was associated with higher hospital mortality.

Epidemiological data show a strong correlation between low VD status and an increased risk of chronic inflammatory diseases, including systemic lupus erythematosus, rheumatoid arthritis, and multiple sclerosis [15].

These findings imply that VD supplements may reduce overall mortality rates; however, more investigation is required to ascertain the precise relationship between baseline VD levels, VD supplement dosage, and overall mortality rates.

Despite the continued rise in interest in VD and the consequent increasing need to measure the levels of total 25-OH-VD (D2/D3) in the blood [19,20,21], some inter-methodological variability, particularly between immunoassays and LC-MS, can still lead to difficulties in the interpretation of analytical results. Therefore, the promotion of laboratory assay standardization, which is essential to achieving results that are comparable across different manufacturers and procedures, has been a major effort to solve this problem [22,23].

In recent years, there has been a significant increase in the number of available methods for measuring VD levels, both LC-MS/MS and immunoassay-based [24,25,26,27,28], increasing the availability of VD testing across both clinical and research settings. While LC-MS/MS remains the gold standard due to its high accuracy and ability to distinguish between different VD metabolites, modern immunoassays have also evolved, offering faster and more automated solutions that are suitable for routine laboratory use.

The Fluorescence Immunoassay (FIA) AFIAS^®^ VD Neo, as described in the Instruction For Use (IFU) [29], may be used to quantitatively measure the levels of total 25-OH-VD (D2/D3) in human whole blood, serum, and plasma.

We had previously evaluated the performance of this diagnostic tool on plasma samples compared to the gold standard, and observed clinically acceptable results [30]. Nevertheless, analysis of plasma requires the separation of corpuscular elements. This pre-analytical step is difficult to perform if you want to use the device as a Point-Of-Care Test (POCT), since this requires sample centrifugation or filtration; therefore, providing a precise and accurate measurement on whole blood would be a great advantage for a POCT. This approach is associated with some advantages, since conducting the test in close proximity to patients and with an extremely short Turn-Around-Time (TAT) can improve patient satisfaction and engagement, allowing the application of immediate therapeutic measures (e.g., VD supplementation), together with reducing costs and risks related to sample transport [31,32,33].

For these reasons, in this work, we cross-validated the analytical performance of a new point-of-care fluorescence immunoassay on whole blood samples, based on a single cartridge with a lateral-flow architecture that has small wells pre-filled with all test reagents (“all-in-one” concept), by comparison with the gold standard LC-MS/MS method, performed with an IVD-marked bioanalytical kit.

## 2. Results

In this work, a VD test method comparison between AFIAS-1^®^ (Boditech AFIAS-1^®^ Immunoassay Analyzer, VD Neo, and the gold standard quantitation method LC-MS/MS (with a commercial VD Kit (Eureka Lab division, Sentinel CH. SpA, Milan, Italy), through the analysis of 50 matched whole blood (for AFIAS-1^®^) and plasma (for LC-MS/MS) samples.

The LC-MS/MS method’s performance in terms of accuracy, precision, linearity, peak shapes, and retention times was already described in a previous work published by Palermiti et al. [30]; although 25-OH-VD includes both 25-OH-VD2 and 25-OH-VD3, the presented data do not include 25-OH-VD2, since this was not detected in any sample, most likely as a result of patients’ limited 25-OH-VD2 intake (oral supplements in Italy only include VD3).

The mean and standard deviation values for AFIAS-1^®^ whole blood and LC-MS/MS plasma measurement are reported in Table 1.

When comparing the LC-MS/MS and AFIAS-1^®^ techniques, Spearman’s correlation coefficient was 0.906 with a *p*-value < 0.0001.

The Anova non-parametric test was used to confirm the homogeneity of variances on AFIAS-1^®^ and LC-MS/MS, reporting a *p*-value of 0.337, indicating that data dispersion was homogeneous between AFIAS-1^®^- and LC-MS/MS-derived results.

Following VD quantification with both methods, Passing–Bablok regression was performed to assess the degree of concordance in results between the two quantitative approaches.

**Figure 1:**
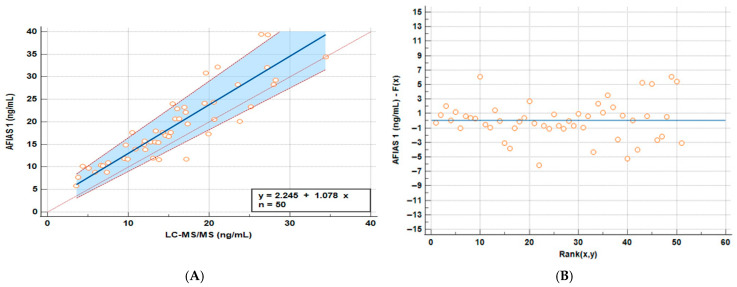
Passing–Bablok (**A**) regression line and the identity line indicating that the two methods to visualize the differences and residuals graph (**B**) graphs comparing whole blood AFIAS-1^®^ and plasma LC-MS/MS samples.

The regression graph comparing whole blood AFIAS-1^®^ with plasma LC-MS/MS quantitation is reported in Figure 1A, while in Figure 1B, it is possible to observe a scatter plot graph of residuals of the Passing–Bablok analysis. All the results on the Regression and Passing–Bablok analysis are reported in Table 2.

The Cusum Test was performed to test the linearity of the different models, and no significant deviation from linearity was observed (*p* = 0.65).

**Figure 2:**
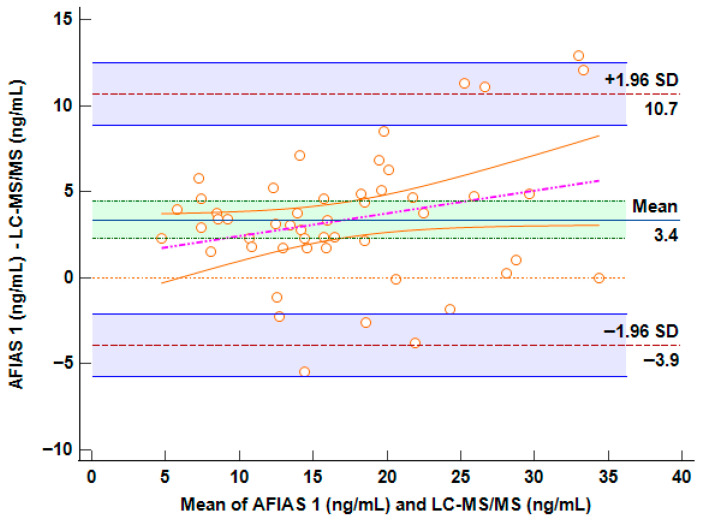
Bland–Altman plot comparing AFIAS-1^®^ versus LC-MS/MS method. The horizontal center line in the Bland–Altman plot represents the mean of the differences (bias) between methods.

Bland–Altman analysis was performed in order to assess whether LC-MS/MS- and AFIAS-1^®^-derived arrangements of data revealed differences between the methods and their agreement in relation to the concentrations, aiming to understand whether the differences found were proportional to the detected concentration (the plot is reported in Figure 2). This test evaluates not only the correlation, but also the actual difference between the measured values; The horizontal center line in the Bland–Altman plot represents the mean of the differences (bias) between methods, indicating whether one method tends to overestimate or underestimate compared to the other. If it is close to 0, on average, the two methods agree.

Approximately 95% of the points should fall within these limits if the differences are normally distributed.

## 3. Discussion

The scientific community has been paying more attention to the role of VD in recent years, with indications emerging that, aside from its role in bone and calcium homeostasis, VD supplementation may act as an immunomodulatory agent [34,35,36] with a number of actions on gene expression and an impact on drug metabolism and plasma concentrations [37,38,39]. As a result, VD levels may vary seasonally and affect medication exposure and therapeutic outcomes [38,39,40].

For all these reasons, measuring VD plasma exposure might be a useful clinical practice tool. The purpose of this work is to compare the performance of the gold standard quantitation method, LC-MS/MS, with the AFIAS^®^ fluorescence immunoassay based on one or more cartridges. As a result, the linearity of the VD quantitation was also confirmed. Specifically, the calibration curve and the R^2^ value over 0.999, as required by FDA guidelines, indicate excellent linearity in the VD quantitation range evaluated when using the Eureka kit [41]. The analyzed samples reported for both the methods show a consistent inter-individual variability for 25-OH-VD; however, the median ± standard deviation of all the techniques is almost the same, demonstrating the comparability of the results from AFIAS^®^ and LC-MS/MS (Table 1).

Bland–Altman analysis was performed in order to verify whether the LC-MS/MS and AFIAS-1^®^ measurement methods were interchangeable.

The Bland–Altman plot shows limits of agreement (±1.96 SD) that range from −3.9 ng/mL to +10.7 ng/mL (Figure 2). This spread suggests moderate variability between the two methods. While most data points fall within these limits, the range is relatively wide: this potential bias could represent a challenge for clinical decision-making in borderline concentrations. On the other hand, it is evident in the plot that the presence of a regression line and a visible positive slope suggests a proportional bias: the difference between methods increases with higher VD concentrations (>35 ng/mL). This implies that AFIAS-1^®^ may become less accurate at higher levels, potentially overestimating more as concentrations rise. Fortunately, the range of concordance between 10 and 20 ng/mL carries the highest importance for representations of VD deficiency in clinical decision-making.

To better explain this issue, even considering a percentage of overestimation of approximately 3.4 ng/mL with AFIAS-1^®^, a patient with concentrations of approximately 25 ng/mL (measured by HPLC-MS/MS) would be within range in the AFIAS-1^®^ measurement (approximately 28.4 ng/mL), thus not significantly impacting clinical decisions. Conversely, at low concentrations, which are those for which supplementation decisions must be made, the two methods are superimposable.

The Anova test was used in this situation to see if one approach may overestimate the interindividual variability: the observed *p*-value of 0.337 indicated no significant differences in variances for the two considered methods.

Although there is a considerable amount of variation, AFIAS-1^®^ appears to have a fixed overestimation (intercept) of roughly 2.25 ng/mL and an average proportional overestimation of roughly 7% (Figure 1).

The measured values fall within the clinically relevant range of 5–40 ng/mL, and the slight overestimation does not significantly affect clinical interpretation. Common therapeutic thresholds are generally >30 ng/mL for broader immune effects and around 20 ng/mL for bone health. Based on the maximum intercept and slope variation, the test may overestimate values by up to +25% (around +3.9 ng/mL) or underestimate by up to −7.5% (around −0.2 ng/mL), each with a probability of about 2.5%. For example, a true value of 13 ng/mL has only a 2.5% chance of being misinterpreted as 20 ng/mL using the AFIAS test and cannot mathematically be read as 30 ng/mL. The probability of misclassification reaches 50% only near the average discrepancy (~+7.8% or +2.25 ng/mL), around 16 ng/mL. Thus, there is a 50% chance of confusing a slightly insufficient level with a borderline sufficient one between 16 and 19.9 ng/mL. However, since the desired optimal vitamin D level is around 30 ng/mL and toxic levels are much higher (>120 ng/mL), this degree of possible misclassification is not clinically meaningful. Even a measured value of 20 ng/mL (which could represent a true level of about 15–16 ng/mL) would still reasonably lead to recommending supplementation. The analysis of the residuals finally highlights that the observed variance is random and not related to high or low concentrations of VD (Figure 1B).

Furthermore, there is a great deal of variation in VD intraindividual concentration, as indicated by [42]. As an exploratory study on a new diagnostic method, the low number of samples (*n* = 50) represents the study’s main limitation, even though it was able to measure VD levels throughout a broad dynamic range of values.

Although the LC-MS/MS method can detect 25(OH)D2, all samples in this study were below the detection limit, likely because patients were supplemented only with 25(OH)D3. Since vitamin D2 supplementation is uncommon in Italy, except among certain groups such as vegans who use plant-based sources, it would be beneficial, in the near future, to expand the comparison to samples from vegan volunteers supplemented with 25(OH)D2 [43]. Additionally, the small and homogeneous cohort limits the assessment of the AFIAS-1^®^ platform across different clinical conditions. Expanding the study to a larger and more diverse population (e.g., osteoporosis patients, those with vitamin D-related disorders, or individuals on supplementation) would allow a more thorough evaluation of clinical performance.

The AFIAS Vitamin Neo All-in-One cartridge has the benefit of allowing the user to take the test without any requirement for additional materials or reagents.

Besides the limitations of this study and the evidenced tendency to overestimate the VD concentrations in the highest ranges, the use of this POCT on whole blood could be beneficial for fast confirmation of VD deficiency or insufficiency, enabling timely decision-making. It is worth noting that 17% and 16% results were measured that fell below 20 ng/mL and 30 ng/mL, respectively, despite AFIAS-1’s slight overestimation, resulting in the clinical decision to supplement.

The Boditech & Menarini AFIAS^®^ family of automated immunofluorescence analyzers, namely AFIAS-1^®^, is ideal for testing at hospital bedsides, doctors’ offices, and pharmacies. When a patient has aberrant levels of calcium, phosphorus, and parathyroid hormone (PTH) (calcium metabolism imbalance), the test findings might be used as evidence to begin aggressive therapy [44] or supplements, such as those that strengthen the immune system [45]. VD deficiency can be diagnosed using this test, which is particularly recommended for people who are at high risk of developing it.

One of the principal advantages of the AFIAS-1^®^ POCT system lies in its markedly reduced turnaround time. The assay delivers quantitative results within approximately 12–15 min, significantly expediting clinical decision-making compared to centralized laboratory methods. Considering the turnaround time of the LC-MS/MS method, the sample management procedure and the preparatory phase require significantly longer times, as well as dedicated equipment and specialized personnel. Using the LC-MS/MS method, reporting could be guaranteed at a minimum of 24 h from the sampling if performed by a highly specialized center, which is significantly longer than the 15 min required by AFIAS-1. This rapidity, coupled with the system’s automated workflow and intuitive interface, renders it highly suitable for decentralized settings such as primary care clinics, pharmacies, and mobile health units. Its compact design and minimal training requirements further enhance its applicability in resource-limited environments, where access to high-complexity instrumentation is often constrained. Compared to plasma, whole blood offers superior sample management since it does not require centrifugation or sample processing time prior to testing, which speeds up the procedure and reduces the possibility of sample operation errors.

To further validate the clinical utility of the AFIAS-1^®^ platform, future research should focus on generating direct comparative data with widely established immunoassays such as electrochemiluminescence immunoassays (ECLIA). Such comparisons are essential not only to assess the analytical performance but also to determine the diagnostic concordance and potential limitations of AFIAS-1^®^ relative to standard laboratory methods, which will ultimately be critical for supporting its broader adoption in routine diagnostic workflows.

## 4. Materials and Methods

Between 9 December 2024 and 23 December 2024, 51 blood samples (16 male and 35 female) from healthy volunteers were collected from capillary blood sampling: each patient signed an informed consent form. Specifically, finger punctures were performed on the 50 volunteers by collecting a drop of blood that was then immediately analyzed on the AFIAS I system. This performance assessment study was conducted on the AFIAS^®^ VD Neo test, a fluorescent immunoassay for the quantitative determination of the 25-OH-D2/D3 level using AFIAS-1^®^ instrumentation (Boditech & Menarini, Gangwon, Republic of Korea), which is able to test 1 sample at a time, which is comparable with the gold standard LC-MS/MS technology.

**Figure 3:**
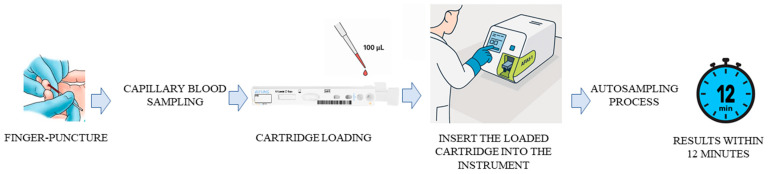
Assay procedure workflow.

Briefly, the test procedure consisted of taking 100 µL of the sample with a pipette and dispensing it into the sample well on the cartridge. Once the cartridge was inserted into its holder and a tip was inserted into the tip hole of the cartridge, the results were displayed on the screen after 12 min (Figure 3). The instrument automatically calculated the test results and displayed the total concentration of 25-hydroxiVD (D2/D3) of the sample in UI/mL (Operating range: 5–100 ng/mL).

Information about sample handling, assay calibration, and quality controls is available upon request from the manufacturing company. Repeatability, precision, and recovery data concerning AFIAS VD Neo have been previously tested and reported maximum CV% as follows: measurement repeatability at different Vit D concentrations reached a maximum CV% of 12.9%; within-laboratory precision of 12.63%; and a multi-site study reproducibility of 11.4%. The accuracy of 3 different lots of AFIAS VD Neo was tested by measuring 10 times at each concentration of the control standard, and all the recovery values did not exceed the value of 105.5%.

As stated in the manufacturer’s Instructions for Use (IFU), the AFIAS-1^®®^ VD assay demonstrates solid analytical sensitivity. Specifically, the reported Limit of Blank (LoB) is 1.888 ng/mL, while the Limit of Detection (LoD) is 3.063 ng/mL. The Limit of Quantification (LoQ) is established at 5.0 ng/mL. These values, provided directly by the company based on internal validation studies, define the assay’s capability to reliably detect and quantify low concentrations of 25-OH-VD.

An aliquot of blood was also collected from the volunteers through Microvette systems in Lithium Heparin, which were centrifuged, and from which approximately 100 microliters of plasma were collected, which was immediately frozen at −80 °C.

In February 2025, a VD kit for LC-MS/MS was acquired (Eureka Lab division, a Sentinel diagnostic company, Sentinel CH. SpA, Milan, Italy), according to all the requirements of Directive 98/79/EC concerning in vitro diagnostic medical devices (IVD), and the declaration of conformity is available upon request.

The EUREKA LC-MS/MS Vit D kit has already been tested for its performance in terms of accuracy, precision, and reproducibility by our laboratory (following EMA and FDA guidelines), in a previously published article, and this method was also evaluated for mutual concordance with the IVD COBAS Vit D immunoassay platform [46].

Briefly, the sample preparation was based on a protein precipitation protocol following the protocol provided by the kit specifications, including internal standardization with a d3-25-OH-VD3. The LC-MS/MS instrument was a LX-50^®^ UHPLC coupled with a QSight 220^®^ (Perkin Elmer, Milan, Italy), and the chromatographic separation was performed in reverse phase on an Acquity^®^ BEH C18 1.7 um 2.1 × 50 mm column (Waters, Milan, Italy) with a gradient of water and methanol both acidified at 0.1% with formic acid, as previously described [30,46].

Plasma samples were thawed and analyzed with the reference method in the Laboratory of Clinical Pharmacology and Pharmacogenetics (University of Turin) on a PerkinElmer LC-MS/MS system (QSight 220). The concordance between the two methods was evaluated by Passing–Bablok regression, considering that the two methods can both be associated with possible errors. In the same way, the homoscedasticity of the residues was described graphically by the plot of the residues.

The quality of fit of the linear model can be visually evaluated based on the residual plot. Because the method assumes a linear link, the residuals should have a random pattern and be close to a normal distribution. If the residuals exhibit a specific pattern, it is expected that the two variables will not have a linear connection.

After accounting for proportional and systematic variations, the residuals show the remaining variance. The interval ±1.96 times the residual standard deviation should contain 95% of the residuals. The random differences between the two laboratory procedures are then defined by this interval (a statistic that is sometimes overlooked when employing Passing–Bablok regression). All the statistical analyses were evaluated through the MedCalc calculator (Version 22.0).

## 5. Conclusions

In conclusion, despite the sample size (*n* = 50) being the minimum necessary to perform methodological comparison evaluations and the absence of high Vit. D values (>40 ng/mL) due to the period (autumn–winter) of sample collection, we observed a good concordance between the methods, which was statistically proven. The AFIAS-1^®®^ system shows strong potential for clinical application, particularly in real-world settings such as outpatient clinics, primary care practices, and other decentralized environments; future studies should aim to provide direct comparative data with widely adopted immunoassays, such as ECLIA, to further support its integration into routine diagnostics. Concluding, the methods evaluated are superimposable, and the minimal potentially observable differences should not have a negative impact from a clinical point of view.

## Figures and Tables

**Table 1:** Mean VD concentrations and inter-individual variability (as standard deviations and CV%) observed in our sample population.

	AFIAS-1^®^ (ng/mL)	LC MS/MS (ng/mL)-Thawed
**Lowest value**	5.86	3.57
**Highest value**	39.42	34.41
**VD3 (mean ± SD)**	18.86 ± 8.18	15.49 ± 7.21
**CV%**	43%	47%

**Table 2:** Regression and Passing–Bablok statistical results. CI = confidence interval.

Regression Equation	
y = 2.244520 + 1.078287 x	
**Systematic differences**	
Intercept A	2.2445
95% CI	−0.2222 to 3.9455
**Proportional differences**	
Slope B	1.0783
95% CI	0.9248 to 1.2553
**Random differences**	
Residual Standard Deviation (RSD)	2.5795
±1.96 RSD Interval	−5.0559 to 5.0559

## Data Availability

Data are contained within the article.
